# Volatile Organic Compounds Produced by a Deep-Sea Bacterium Efficiently Inhibit the Growth of *Pseudomonas aeruginosa* PAO1

**DOI:** 10.3390/md22050233

**Published:** 2024-05-20

**Authors:** Yuanyuan Hu, Ge Liu, Chaomin Sun, Shimei Wu

**Affiliations:** 1 College of Life Sciences, Qingdao University, 308 Ningxia Road, Qingdao 266071, China; yuanyuanhu2023@163.com; 2 CAS Key Laboratory of Experimental Marine Biology & Center of Deep Sea Research, Institute of Oceanology, Chinese Academy of Sciences, Qingdao 266071, China; liuge@qdio.ac.cn; 3 Laboratory for Marine Biology and Biotechnology, Qingdao Marine Science and Technology Center, Qingdao 266071, China; 4 Center of Ocean Mega-Science, Chinese Academy of Sciences, Qingdao 266071, China; 5 College of Earth Science, University of Chinese Academy of Sciences, Beijing 101408, China

**Keywords:** antibacteria, volatile organic compounds, *Pseudomonas aeruginosa* PAO1, cell division, biofilm

## Abstract

The deep-sea bacterium *Spongiibacter nanhainus* CSC3.9 has significant inhibitory effects on agricultural pathogenic fungi and human pathogenic bacteria, especially *Pseudomonas aeruginosa*, the notorious multidrug-resistant pathogen affecting human public health. We demonstrate that the corresponding antibacterial agents against *P. aeruginosa* PAO1 are volatile organic compounds (VOCs, namely VOC-3.9). Our findings show that VOC-3.9 leads to the abnormal cell division of *P. aeruginosa* PAO1 by disordering the expression of several essential division proteins associated with septal peptidoglycan synthesis. VOC-3.9 hinders the biofilm formation process and promotes the biofilm dispersion process of *P. aeruginosa* PAO1 by affecting its quorum sensing systems. VOC-3.9 also weakens the iron uptake capability of *P. aeruginosa* PAO1, leading to reduced enzymatic activity associated with key metabolic processes, such as reactive oxygen species (ROS) scavenging. Overall, our study paves the way to developing antimicrobial compounds against drug-resistant bacteria by using volatile organic compounds.

## 1. Introduction

*Pseudomonas aeruginosa* is a widespread pathogen found in various environments including aqueous environments and the infected tissues of human beings. *P. aeruginosa* poses a significant challenge to global human health as it can lead to both acute and chronic infections [1]. In patients with lung diseases, particularly those with cystic fibrosis, colonization of the lungs by *P. aeruginosa* is a major contributor to illness and mortality [2]. Notably, research indicates that this infection is associated with the formation of highly antibiotic-resistant biofilms [3]. Biofilm formation in *P. aeruginosa* is governed by intricate bacterial regulatory systems, which include quorum sensing (QS) systems reliant on cell density and intercellular communication [1]. QS systems in *P. aeruginosa* play a crucial role in regulating the release of virulence factors and influencing iron uptake processes [4]. Therefore, the inhibition of QS systems has garnered considerable interest, including quorum quenching (QQ), which involves the use of chemical or enzymatic means to inhibit QS systems, thereby attenuating behaviors regulated by QS systems [5]. QS systems regulate the expression of a series of key genes by sensing signaling molecules called autoinducers. As cell density increases, autoinducers accumulate in the extracellular environment. When the concentration of the autoinducer reaches a certain threshold, QS systems are activated [6]. The activation of QS systems relies on the essential process of cell division, which not only facilitates bacterial population growth but also aids in the transmission of genetic information within *P. aeruginosa* [7]. Cell division in bacteria is mediated by the cytokinesis complex, which consists of the eukaryotic microtubule protein homolog FtsZ self-assembling into dynamic protofilaments and serving as scaffolds for cell division and dozens of other related proteins [8]. The division complex promotes cell division through a series of processes, mainly including cell shrinking as well as synthesis of the septal peptidoglycan (sPG) wall [9]. The synthesis of sPG during bacterial cell division is a crucial and highly conserved process that is essential for reproduction, which makes division proteins intriguing targets in the search for novel antibiotics.

Searching for novel antibiotics against *P. aeruginosa* is always a fascinating direction in both microbiological and biomedical fields. Recently, many drug candidates targeting different essential growth processes of *P. aeruginosa* have been reported, such as exopolysaccharide EPS273 [10], difficidin analogs [11], and pyran polyketide penipyranicins A–C [12]. In addition to non-volatile natural products, volatile organic compounds (VOCs) are increasingly recognized as promising bioactive molecules due to their wide range of ecological functions [13]. Biological VOCs are produced by plants [14], fungi [15], and bacteria [16]. Microbial VOCs have been reported to be by-products produced by microorganisms during primary metabolism or secondary metabolism [17]. Several studies have indicated that microbial VOCs exhibit inhibitory effects on *P. aeruginosa* [18,19]. Nevertheless, the mechanisms by which VOCs inhibit *P. aeruginosa* have not been deeply elucidated due to their small molecular weight and volatile characteristics [20]. Importantly, VOCs also broadly exist in aquatic environments. Research suggests that the production of VOCs can suppress other microorganisms within the same ecological niche, thereby providing ecological advantages to the strain. Additionally, VOCs enable predators to detect nearby prey based on specific VOC signatures [21,22].

The ocean, being the largest aquatic environment on Earth, presents an ideal setting for the search for novel VOCs. Additionally, the deep sea represents a unique habitat where microorganisms have developed distinct metabolic mechanisms in adaptation to extreme conditions of high pressure, low temperature, and other specific environmental factors [23]. The primary or secondary metabolites generated by these microorganisms in their life processes serve as a valuable resource for the discovery of novel VOCs. Indeed, VOCs produced by a deep-sea bacterium *Bacillus aryabhattai* MCCC 1K02966 have been demonstrated to possess nematocidal activity [24]. In this study, we find that the volatile compound VOC-3.9, produced by the deep-sea Gram-negative bacterium *Spongiibacter nanhainus* CSC3.9, exhibits strong inhibitory activities against several known agricultural pathogenic fungi and the human pathogenic bacterium *P. aeruginosa* PAO1. In addition, the relevant antimicrobial mechanisms were systematically revealed.

## 2. Results and Discussion

### 2.1. The Deep-Sea Bacterium Spongiibacter Nanhainus CSC3.9 Produces Volatile Organic Compounds with Antibacterial and Antifungal Activities

**Figure 1:**
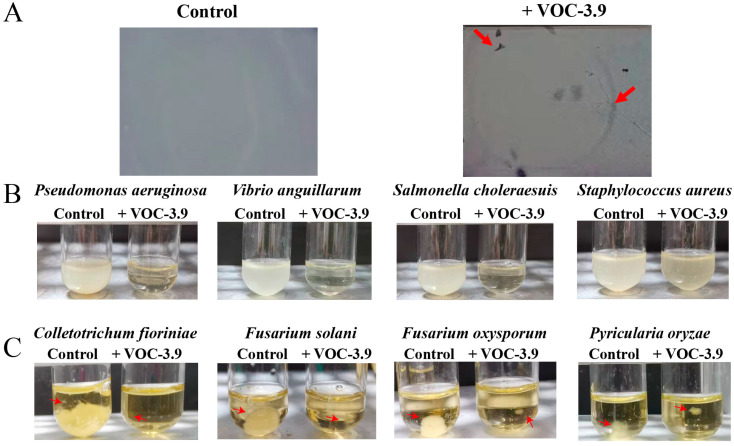
VOC-3.9 exhibits broad-spectrum inhibitory effects against various pathogenic bacteria and fungi. (**A**) VOC-3.9 possesses a prominent inhibitory effect against *Pseudomonas aeruginosa* PAO1 tested by a modified bioautography method. A total of 60 μL of sterile water and VOC-3.9 are added to silica gel plates for the control group and experimental group, respectively. The plates are then coated with LB solid medium containing pre-cultured *P. aeruginosa* PAO1 cells and incubated at 37 °C for 24 h. The presence or absence of antibacterial zones on the plates is visually observed. (**B**) VOC-3.9 displays obvious inhibitory effects on Gram-negative bacteria (including *P. aeruginosa* PAO1, *Vibrio anguillarum*, and *Salmonella choleraesuis*) at a concentration of 2.5 mg mL^−1^, cultured for 24 h at 37 °C or 28 °C, and a slight inhibitory effect on the Gram-positive bacterium *Staphylococcus aureus*. (**C**) VOC-3.9 exerts evident inhibitory effects on agricultural fungal pathogens, including *Colletotrichum fioriniae*, *Fusarium solani*, *Fusarium oxysporum*, and *Pyricularia oryzae*.

In 2017, the World Health Organization designated *P. aeruginosa* as the top priority among drug-resistant bacteria in the quest for new antibiotics [25]. Consequently, we consistently used *P. aeruginosa* PAO1 as the model bacterium to screen for relevant active strains throughout the experiment. In this study, we discovered that the deep-sea bacterium *Spongiibacter nanhainus* CSC3.9 synthesized bioactive compounds that effectively inhibited the growth of *Pseudomonas aeruginosa* PAO1 through screening. *S. nanhainus* CSC3.9, a Gram-negative bacterium isolated from deep-sea cold seeps, has been shown to possess the ability to sense blue light in our recent study [26]. However, inhibitory activity was only detected in the liquid medium but not in the agar plate tested by the traditional agar diffusion method. Moreover, none of the retained substances exhibited inhibitory activity when we tried to concentrate the active fraction using freeze-drying and vacuum- spinning methods. Based on our experiences and previous reports [27,28,29], we speculated that the antibacterial substance produced by *S. nanhainus* CSC3.9 might be volatile. Therefore, we vaporized the original antibacterial substances by using a rotary evaporator under vacuum at 45 °C and collected the distilled fractions. Indeed, the distilled parts showed an evident inhibitory effect on the growth of *P. aeruginosa* PAO1 on the silica gel plates by using a modified bioautography method (Figure 1A), which was usually utilized to detect the activity of volatile organic compounds (VOCs) [30,31]. The above results clearly indicated that the antibacterial substances produced by *S. nanhainus* CSC3.9 were VOCs, and we named them VOC-3.9 in this study.

In order to measure the antibacterial spectrum of VOC-3.9, various bacteria and fungi were selected. In addition to *P. aeruginosa* PAO1, VOC-3.9 also effectively inhibited the growth of other Gram-negative pathogenic bacteria such as *Vibrio anguillarum* and *Salmonella choleraesuis*. It is known that *S. choleraesuis* are common human- and food-pathogenic bacteria, and their antibiotic resistance is increasing year by year [32]. *V. anguillarum* is a pathogenic bacterium that severely impacts the aquaculture industry, causing infections such as fish hemorrhagic septicemia and vibriosis [33]. VOC-3.9 slightly inhibited the Gram-positive pathogenic bacterium *Staphylococcus aureus* (Figure 1B), and evidently inhibited four agricultural pathogenic fungi (including *Colletotrichum fioriniae*, *Fusarium solani*, *Fusarium oxysporum*, and *Pyricularia oryzae*) (Figure 1C). For the four fungal pathogens, *C. fioriniae* is an important plant pathogenic, saprophytic, and endophytic fungus that causes rot in important crops such as strawberries, apples, blueberries, and pears [34]; *F. solani* can cause root rot in a wide range of economically important crops such as citrus, vegetables, flowers, field crops worldwide [35], and even humans and animals [36]; *F. oxysporum* is a pathogen that affects cucumbers [37]; *P. oryzae* is an important pathogen that causes rice blast disease [38]. Therefore, the above results suggested that VOC-3.9 had a broad inhibitory spectrum and could be developed as a potential antibiotic against both bacterial and fungal pathogens in the medical and agricultural fields.

### 2.2. Identification of Active Compounds against P. aeruginosa PAO1 within VOC-3.9

**Figure 2:**
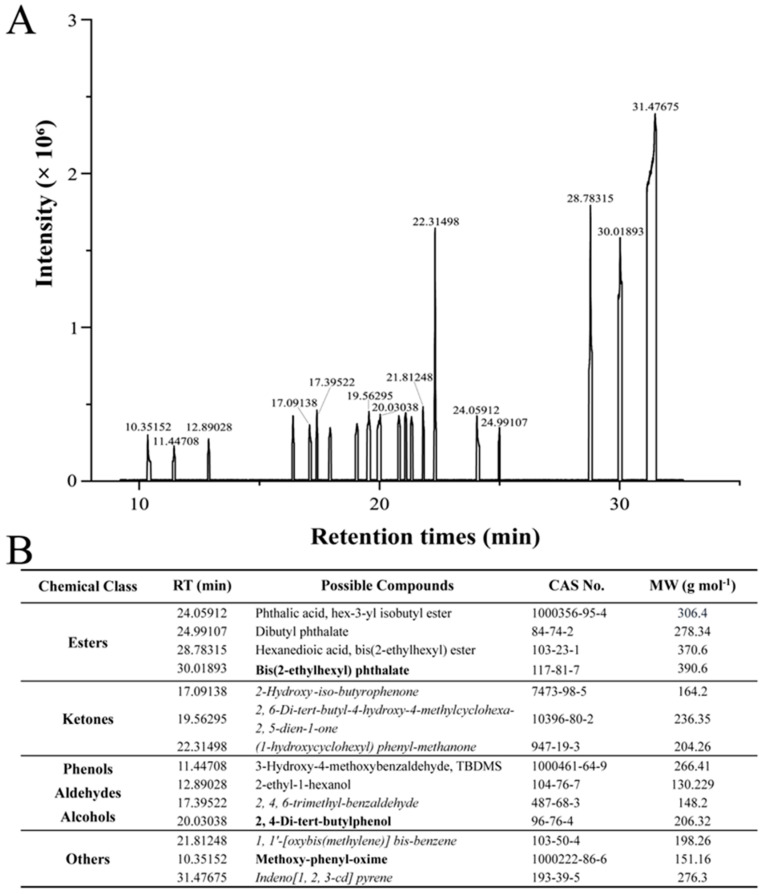
Determination of the composition of VOC-3.9 through gas chromatography-mass spectrometry (GC-MS). (**A**) Qualitative analysis of the components of VOC-3.9 using solid-phase microextraction (SPME) coupled with GC-MS. (**B**) Detail information of 14 identified volatile organic compounds within VOC-3.9 based on the GC-MS results shown in panel A. RT indicates retention times; CAS indicates chemical abstracts service number; MW indicates molecular weight. The compounds that have been previously reported to inhibit *P. aeruginosa* are indicated in bold text. The compounds that have not previously been detected in microorganisms are marked in italics.

Considering that *P. aeruginosa* is one of the most notorious pathogens affecting human public health, our focus was on understanding the inhibitory mechanism of VOC-3.9 against *P. aeruginosa* PAO1. To identify the effective constituents of VOC-3.9 against *P. aeruginosa* PAO1, we further analyzed it using solid-phase microextraction (SPME) coupled with GC-MS (Figure 2A). A total of 14 compounds within VOC-3.9 were identified, including four esters, three ketones, four phenols/aldehydes/alcohols, and three other types of compounds (Figure 2B). Among these 14 compounds within VOC-3.9, eight of them (Phthalic acid, hex-3-yl isobutyl ester; Dibutyl phthalate; Hexanedioic acid, bis(2-ethylhexyl) ester; Bis(2-ethylhexyl) phthalate; 3-Hydroxy-4-methoxybenzaldehyde; 2-ethyl-1-hexanol; 2, 4-Di-tert-butylphenol; Methoxy-phenyl-oxime) have been reported as biological sources, and were shown to have inhibitory effects on fungal, bacterial, or tumor cells [28,39,40,41,42,43,44,45]. To our knowledge, the other six substances (including 2-Hydroxy-iso-butyrophenone; 2, 6-Di-tert-butyl-4-hydroxy-4-methylcyclohexa-2, 5-dien-1-one; (1-hydroxycyclohexyl) phenyl-methanone; 2, 4, 6-trimethyl-benzaldehyde; 1, 1′-[oxybis(methylene)] bis-benzene, and Indeno[1, 2, 3-cd] pyrene) are proposed for the first time as microbial sources in our present study (Figure 2B).

**Figure 3:**
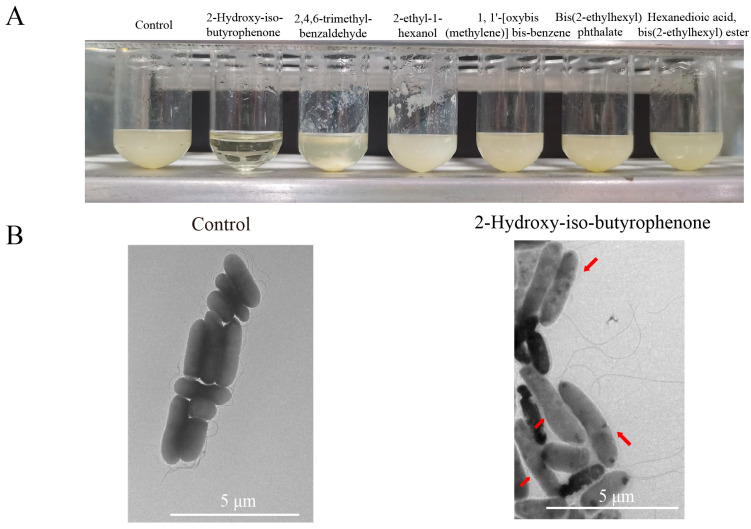
Antibacterial assays of six compounds within VOC-3.9 against *P. aeruginosa* PAO1. (**A**) 2-Hydroxy-iso-butyrophenone within VOC-3.9 shows significant antibacterial activity against *P. aeruginosa* PAO1 at concentrations above 2.4 mg mL^−1^. In comparison, the other five substances within VOC-3.9 at 5 mg mL^−1^ do not show evident inhibitory effects on the growth of *P. aeruginosa* PAO1 cultured at 37 °C for 24 h with a rotation speed of 150 rpm. The same amount of sterile water was added to the culture of *P. aeruginosa* PAO1 and the control. The compound name is indicated above each test tube. (**B**) TEM observation of the effect of 0.3 mg mL^−1^ of 2-Hydroxy-iso-butyrophenone on the morphology of *P. aeruginosa* PAO1 cells cultured at 37 °C for 16 h with a rotation speed of 150 rpm. *P. aeruginosa* PAO1 cells treated with the same amount of sterile water were used as the control.

To further determine which substances within VOC-3.9 indeed perform key roles in inhibiting the growth of *P. aeruginosa* PAO1, six available chemically synthesized VOCs were selected for activity assays. Due to the volatilization of VOC-3.9, we chose a higher concentration for the activity assay. The results showed that 2-Hydroxy-iso-butyrophenone; 2, 4, 6-trimethyl-benzaldehyde, and 2-ethyl-1-hexanol could inhibit the growth of *P. aeruginosa* PAO1 to some extent at a concentration of 5 mg mL^−1^ for 24 h at 37 °C with a rotation speed of 150 rpm (Figure 3A). Among them, 2-Hydroxy-iso-butyrophenone showed the strongest inhibiting activity. In addition, 2-Hydroxy-iso-butyrophenone completely inhibits the growth of *P. aeruginosa* PAO1 at a concentration of 2.4 mg mL^−1^. Given the volatile characteristics of 2-Hydroxy-iso-butyrophenone, its working concentration in nature should be much lower than 2.4 mg mL^−1^. As far as we know, 2-Hydroxy-iso-butyrophenone is the first reported microbial VOC against *P. aeruginosa* PAO1. We further treated *P. aeruginosa* PAO1 with 2-Hydroxy-iso-butyrophenone at a concentration of 0.3 mg mL^−1^ for 16 h at 37 °C with a rotation speed of 150 rpm and observed the cell morphological changes using TEM. The cells of *P. aeruginosa* PAO1 became stretched (Figure 3B), suggesting 2-Hydroxy-iso-butyrophenone significantly disordered the growth of *P. aeruginosa* PAO1 and should be one of the essential antibacterial components within VOC-3.9. Importantly, three other substances within VOC-3.9 (including Bis(2-ethylhexyl) phthalate; 2, 4-Di-tert-butylphenol, and Methoxy-phenyl-oxime) have been shown to possess potential inhibitory activities against *P. aeruginosa* [42,44,45]. Among them, Bis(2-ethylhexyl) phthalate was produced by the actinomycete strain GRG4 and inhibited *P. aeruginosa* by altering the integrity of the cell membrane and content efflux, leading to cell death [42]; 2,4-Di-tert-butylphenol was produced by the fungal strain *Diaporthe phaseolorum* SSP12 and hindered the quorum sensing (QS) systems of *P. aeruginosa* PAO1 [44]; methoxy-phenyl-oxime was produced by the bacterium *Klebsiella pneumoniae* and showed potential anti-biofilm activity against *P. aeruginosa* [46]. Collectively, we speculated that VOC-3.9 should contain several effective components against *P. aeruginosa* PAO1 by acting on various targets, and some of them might have a cooperative relationship. In the future, much effort should be exhibited to clearly disclose the exact structure and function of each component within VOC-3.9.

### 2.3. VOC-3.9 Disorders the Cell Division Process of P. aeruginosa PAO1

**Figure 4:**
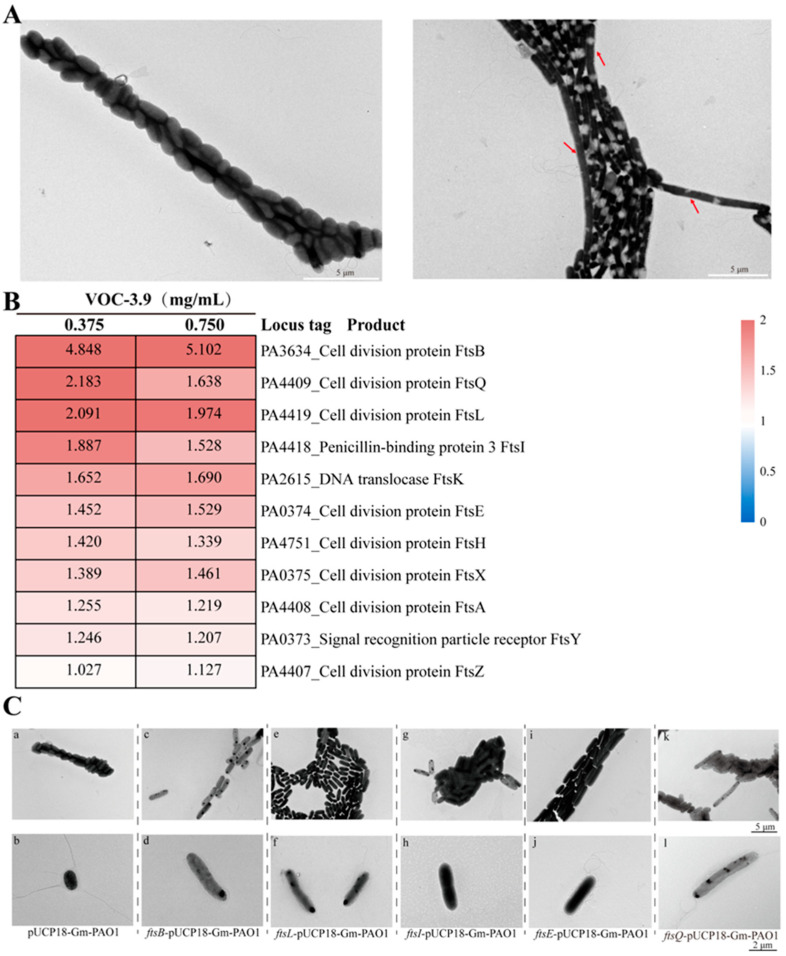
Microscopic and proteomic analyses of the effect of VOC-3.9 on the cell division process of *P. aeruginosa* PAO1. (**A**) TEM observation of the morphology of *P. aeruginosa* PAO1 cells that were cultured in the presence of 0.3 mg mL^−1^ VOC-3.9. *P. aeruginosa* PAO1 cells cultured in medium supplemented with the same amount of sterile water are used as the control. The bacterial cells were cultured at 37 °C for 16 h with a rotation speed of 150 rpm. The red arrows indicate cells of *P. aeruginosa* PAO1 with significant morphological changes. (**B**) Proteomic assays of *P. aeruginosa* PAO1 cells that, respectively, cultured in the presence of 0.375 mg mL^−1^ and 0.75 mg mL^−1^ VOC-3.9 at 37 °C for 16 h with a rotation speed of 150 rpm. In the heatmap, the expression upregulation folds of Fts-related proteins associated with the cell division process compared to those in the control group are indicated. The expressions of corresponding proteins in *P. aeruginosa* PAO1 cells cultured in medium supplemented with the same amount of sterile water are used as the control. (**C**) TEM observation of *P. aeruginosa* PAO1 cells with overexpression of Fts-related proteins. Morphology of *P. aeruginosa* PAO1 cells with the overexpression of FtsB (panels c and d), FtsL (panels e and f), FtsI (panels g and h), FtsE (panels i and j), and FtsQ (panels k and l). Morphology of *P. aeruginosa* PAO1 cells transformed with the empty expression vector is used as the control (panels a and b).

To better understand the inhibitory mechanism of VOC-3.9 against *P. aeruginosa* PAO1, we treated *P. aeruginosa* PAO1 cultures with 0.3 mg mL^−1^ VOC-3.9 for 16 h at 37 °C with a rotation speed of 150 rpm, and then observed bacterial cells with TEM. Compared to the control cells, the cell size of VOC-3.9-treated *P. aeruginosa* PAO1 exhibited significant elongation (Figure 4A), even longer than those treated by 2-Hydroxy-iso-butyrophenone alone (Figure 3B), indicating some other compounds within VOC-3.9 also possess antibacterial activities. To explore the antibacterial mechanism of VOC-3.9 against *P. aeruginosa* PAO1, we, respectively, treated *P. aeruginosa* PAO1 with 0.375 mg mL^−1^ and 0.75 mg mL^−1^ VOC-3.9, and performed proteomic analysis at 37 °C for 16 h with a rotation speed of 150 rpm. Treatment with an equivalent amount of sterile water was used as the control. The proteomic results showed that VOC-3.9 led to significantly upregulated expressions of proteins involved in the cell division process of *P. aeruginosa* PAO1 (Figure 4B). This result was consistent with the TEM observation results (Figure 4A), indicating that the cell division process of *P. aeruginosa* PAO1 was evidently affected by VOC-3.9. The upregulated proteins included those associated with septal peptidoglycan (sPG) synthesis (FtsW and FtsI) and the activation of sPG synthesis (FtsB, FtsQ, and FtsL) [9], as well as peptidoglycan hydrolysis (FtsE and FtsX) [47]. Clearly, based on both microscopic and proteomic results, the cell division process of *P. aeruginosa* PAO1 was disordered by VOC-3.9, especially through upregulating some essential Fts factors involved in cell division.

Next, we overexpressed five genes (including *ftsB*, *ftsL*, *ftsI ftsE*, and *ftsQ*) in *P. aeruginosa* PAO1 that were most significantly upregulated by VOC-3.9 and checked the corresponding effects on the bacterial cell division process. The empty vector used for overexpression was transformed to *P. aeruginosa* PAO1 and used as the control. The TEM observation results showed that the overexpression of *ftsB*, *ftsL*, *ftsI ftsE*, and *ftsQ* indeed increased the cell length of *P. aeruginosa* PAO1 cells when compared to the control group (Figure 4C), confirming the validity of the proteomic results. These findings suggest that VOC-3.9 disrupts the cell division process of *P. aeruginosa* PAO1 by upregulating the expression of key Fts factors associated with sPG synthesis, resulting in cytokinesis abnormalities. It is known that cell division is a crucial process for bacterial growth and reproduction, and it has always been an important target in the search for antibacterial agents [48]. VOC-3.9, a cell division inhibitor, effectively impedes the rapid proliferation of *P. aeruginosa* PAO1, making it a good candidate for preventing and controlling infectious diseases caused by *P. aeruginosa* PAO1.

### 2.4. VOC-3.9 Impedes the Quorum Sensing (QS) Systems Associated with the Biofilm Formation and Dispersion Processes of P. aeruginosa PAO1

**Figure 5:**
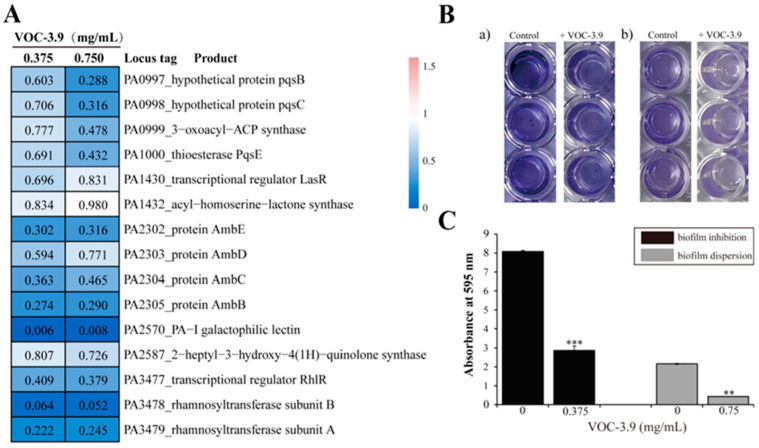
Proteomic and biochemical analyses of the effects of VOC-3.9 on the quorum-sensing systems and associated biofilm formation and dispersion processes of *P. aeruginosa* PAO1. (**A**) Proteomic analysis of the downregulating effects of VOC-3.9 (at 0.375 and 0.75 mg mL^−1^) on the expressions of key proteins related to the quorum sensing (QS) systems of *P. aeruginosa* PAO1. The expressions of corresponding proteins in *P. aeruginosa* PAO1 cells cultured in medium supplemented with the same amount of sterile water are used as the control. (**B**) Observation of the biofilm formation of *P. aeruginosa* PAO1 at 37 °C for 24 h in the absence or presence of 0.375 mg mL^−1^ VOC-3.9 (panel a). Observation of the biofilm dispersion of *P. aeruginosa* PAO1 at 37 °C for 24 h in the absence or presence of 0.75 mg mL^−1^ VOC-3.9 (panel b). In the control group, the same amount of sterile water is added to the culture of *P. aeruginosa* PAO1. (**C**) Quantitative assays of anti-biofilm activity of 0.375 mg mL^−1^ VOC-3.9 and biofilm dispersion activity of 0.75 mg mL^−1^ VOC-3.9 against *P. aeruginosa* PAO1 based on the results shown in panel (**B**). The data were presented as means ± SD of three experiments. ** *p* ≤ 0.01, *** *p* ≤ 0.001.

When analyzing the proteomic results of *P. aeruginosa* PAO1 treated by VOC-3.9, we found that the expressions of many proteins associated with QS systems (e.g., LasR, RhlR, PqsB, and AmbB) were significantly downregulated (Figure 5A). It is well known that both QS systems and biofilm formation/dispersion are closely correlated and are essential for the growth and infection of *P. aeruginosa* PAO1 [49]. We thus checked the inhibitory effect of VOC-3.9 on the biofilm formation of *P. aeruginosa* PAO1 by using 96-well polystyrene plates. The results showed that biofilm formation was markedly inhibited (Figure 5B, panel a) and biofilm dispersion was significantly promoted (Figure 5B, panel b) in *P. aeruginosa* PAO1 by the supplement of 0.375 mg mL^−1^ and 0.75 mg mL^−1^ VOC-3.9, respectively. The biofilm inhibition ratio and biofilm dispersion ratio of *P. aeruginosa* PAO1 could, respectively, reach 64.56% and 79.99% by the treatment of VOC-3.9 (Figure 5C). QS systems are critical in regulating the generation of virulence factors in *P. aeruginosa* PAO1 [50], and the biofilm is a good target for developing novel antibiotics and avoiding drug resistance [10]. Obviously, VOC-3.9 effectively inhibits the QS systems associated with biofilm formation and dispersion in *P. aeruginosa* PAO1 (Figure 5). These results demonstrate the potential of VOC-3.9 as a drug candidate with anti-biofilm activities that can lower antibiotic resistance against *P. aeruginosa* PAO1.

### 2.5. VOC-3.9 Hinders Iron Uptake in P. aeruginosa PAO1

**Figure 6:**
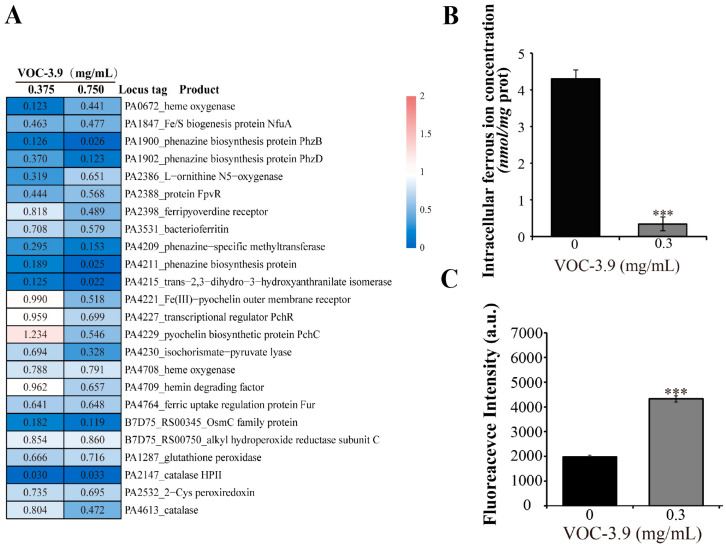
Proteomic and biochemical analyses of the effects of VOC-3.9 on the iron uptake process and ROS accumulation of *P. aeruginosa* PAO1. (**A**) Proteomic analysis of the downregulating effects of VOC-3.9 (at 0.375 and 0.75 mg mL^−1^) on the expressions of key proteins related to iron uptake, phenazine synthesis, and reactive oxygen species (ROS)-scavenging enzyme of *P. aeruginosa* PAO1. The expressions of corresponding proteins in *P. aeruginosa* PAO1 cells cultured in medium supplemented with the same amount of sterile water are used as the control. (**B**) Detection of intracellular ferrous ion concentrations of *P. aeruginosa* PAO1 treated with 0.3 mg mL^−1^ of VOC-3.9 at 37 °C for 16 h with a rotation of 150 rpm, respectively. The intracellular ferrous ion concentration of *P. aeruginosa* PAO1 treated with the same amount of sterile water is used as the control. (**C**) Determination of intracellular ROS levels of *P. aeruginosa* PAO1 treated with 0.3 mg mL^−1^ VOC-3.9 at 37 °C for 16 h with a rotation of 150 rpm, respectively. The intracellular ROS accumulation level of *P. aeruginosa* PAO1 treated with the same amount of sterile water is used as the control. The data were presented as means ± SD of three experiments *** *p* ≤ 0.001.

Iron is an essential cofactor of several core enzyme systems of *P. aeruginosa* PAO1 and also plays a key role in biofilm formation in *P. aeruginosa* PAO1. To obtain extracellular iron, *P. aeruginosa* PAO1 secretes high-affinity siderophores and heme to bind extracellular Fe^3+^, which is subsequently transported across the cell membrane and ultimately reduced to Fe^2+^ [51]. Interestingly, the proteomic results showed that the expressions of many key genes associated with iron uptake (e.g., *pchR*, *fpvA* and *phuT*) and reduction processes (e.g., PA4708) of *P. aeruginosa* PAO1 were evidently downregulated (Figure 6A). Indeed, the intracellular ferrous ion concentrations were markedly decreased when *P. aeruginosa* PAO1 was treated with 0.3 mg mL^−1^ VOC-3.9 at 37 °C for 16 h with a rotation speed of 150 rpm (Figure 6B), confirming that the iron uptake processes of *P. aeruginosa* PAO1 were hindered by supplementation with VOC-3.9. A previous study has shown that QS systems and iron uptake processes interact with each other and cooperatively regulate the expression of various genes including virulence factors [52].

On the other hand, iron is an essential cofactor for several core enzyme systems, including peroxidase and catalase, associated with reactive oxygen species (ROS) scavenging [53]. Excess intracellular ROS can react with bacterial lipids, DNA, and proteins, resulting in lipid peroxidation and gene mutations [54]. Indeed, exposure to VOC-3.9 resulted in a significant decrease in the expressions of some peroxidases and catalases (Figure 6A), and an obvious increase in the intracellular ROS concentration of *P. aeruginosa* PAO1 (Figure 6C). The results indicate that the treatment of *P. aeruginosa* PAO1 with VOC-3.9 could result in an elevation in the amount of ROS, thereby leading to further cell damage.

## 3. Materials and Methods

### 3.1. Bacterial Strains and Culture Conditions

*Spongiibacter nanhainus* CSC3.9 was previously isolated from deep-sea cold seeps [26]. *S. nanhainus* CSC3.9 and *Vibrio anguillarum* were cultured in 2216E medium (containing 5 g L^−1^ tryptone, and 1 g L^−1^ yeast extract in a liter of filtered seawater with pH adjusted to about 7.4; Oxoid, Basingstoke UK). Apart from *V. anguillarum*, other pathogenic bacteria (including *Pseudomonas aeruginosa* PAO1, *Staphylococcus aureus*, and *Salmonella choleraesuis*) were cultured in Luria Bertani (LB) medium (containing 10 g L^−1^ tryptone, 5 g L^−1^ yeast extract, and 10 g L^−1^ NaCl in a liter of distilled water with pH adjusted to 7.0; Oxoid, Basingstoke, UK) at 37 °C. The pathogenic fungal strains (including *Colletotrichum fioriniae*, *Fusarium solani*, *Fusarium oxysporum*, and *Pyricularia oryzae*) were inoculated onto potato dextrose agar (PDA) medium (containing 200 g L^−1^ potato, 20 g L^−1^ glucose, and 15 g L^−1^ agar in a liter of distilled water; Solarbio, Beijing, China). The cultures were incubated at 28 °C. The final gentamicin concentration used for assays was 25 μg mL^−1^.

### 3.2. Collection of Volatile Organic Compounds (VOCs)

To obtain VOCs, the supernatant of *S. nanhainus* CSC3.9 cultured in 2216E medium for 7 days was subjected to alcoholic precipitation. Then, the precipitate was solubilized using ultrapure water and boiled for 30 min to remove proteins. The solution was centrifuged at 8000× *g* for 20 min. Subsequently, the fractions with higher molecular weights were removed using an ultrafiltration tube with a molecular retention capacity of 3000 Da. Finally, the fractions were collected by vaporization using a rotary evaporator under vacuum at 45 °C to obtain the gas chromatography-mass spectrometry (GC-MS) sample, named VOC-3.9.

### 3.3. Activity Test of VOC-3.9

For activity assays of VOC-3.9, the traditional TLC-bioautography method was performed with minor modifications [30,31]. Briefly, 60 μL cell-free supernatant of *S. nanhainus* CSC3.9 was taken as a suspension drop on a 3 × 5 cm sterilized silica gel plate (Jiaoao, Yantai, China), and the same amount of sterile water was used as a control. The overnight-cultured *P. aeruginosa* PAO1 was then added to the LB solid medium at 1% inoculum, mixed well, and poured onto the silica gel plates spiked with VOC-3.9 or water. After that, the silica gel plates were incubated at 37 °C for 24 h. After removing the solid medium, the silica gel plates were treated with 3-(4, 5-dimethylthiazol-2-yl)-2, 5-diphenyltetrazolium bromide (MTT, Sigma, Saint Louis, MO, USA) for 30 min.

### 3.4. Antibacterial and Antifungal Assays of VOC-3.9

Briefly, overnight cultured bacterial or fungal cells were used as the seed solution. Bacterial cells were added to LB medium or 2216E medium at a concentration of 1 × 10^5^ CFU (colony forming unit) mL^−1^. Activated, equally sized fungal pellets were added to the PDB medium. In the control group, an equivalent amount of sterile water was added. The cultures of *V. anguillarum* and fungal strains were cultured at 28 °C for 24 h with a rotation speed of 150 rpm. Other bacterial strains were cultured at 37 °C for 24 h with a rotation speed of 150 rpm. Finally, the growth status of different bacterial and fungal strains was visually observed.

### 3.5. Characterization of VOC-3.9 by GC-MS

To identify the components of VOC-3.9, solid-phase microextraction (SPME) coupled with chromatographic tandem mass spectrometric analysis was performed according to the method described previously [55]. The SPME fibers (65 μm, polydimethylsiloxane/divinylbenzene (Supelco, Bedford, MA, USA)) were inserted into headspace vials, which were then exposed to adsorption at the shaking rate of 450 rpm and the temperature of 80 °C for 30 min. The compounds were then analyzed on a GC-MS coupled to an 8890-5977B (Agilent, Santa Clara, CA, USA) in the injection port at 250 °C for 5 min. Helium was used as the carrier gas at a flow rate of 1.2 mL min^−1^ through an HP-5MS column (30 m × 0.25 m × 0.5 μm) (Agilent, Santa Clara, CA, USA). The temperature increase program was set as follows: the initial column temperature was 40 °C, held for 5 min, increased to 320 °C at a rate of 10 °C min^−1^, and finally held for 2 min. The mass spectrometer was used in the electron ionization mode with a source temperature of 230 °C and a quadrupole temperature of 150 °C, and the full scan m/z was used, in the range of 20–500. The mass spectrometry data were computer-matched with compounds contained in Agilent Hewlett-Packard NIST 20 and the Wiley ver. 6 MASS SPECTRAL DATABASE to the mass spectrometry data for volatile peak designation.

### 3.6. Activity Assays of Single Components of VOC-3.9 against P. aeruginosa PAO1

Six chemically produced single substances (including 2-Hydroxy-iso-butyrophenone; 2, 4, 6-trimethyl-benzaldehyde; 2-ethyl-1-hexanol; 1, 1′-[oxybis(methylene)] bis-benzene, Bis(2-ethylhexyl) phthalate; Hexanedioic acid, bis(2-ethylhexyl) ester) within VOC-3.9 were purchased from reagent companies according to the type of reagent. Activity assays of these six substances against *P. aeruginosa* PAO1 were performed as described above. However, due to the lack of methods for detecting inhibitory concentrations of VOCs under sterile and aerobic conditions, we used high concentrations for bacteriostatic activity testing to make sure that activity can be detected. The final concentration for each compound in the test tubes was 2~5 mg mL^−1^. Overnight pure cultured *P. aeruginosa* PAO1 was inoculated into test tubes at a 1% concentration and subsequently incubated at 37 °C for 24 h with a rotation speed of 150 rpm. The inhibitory effect of six substances on *P. aeruginosa* PAO1 was determined through the turbidity of the bacterial suspension. Furthermore, we determined the antimicrobial concentration of 2-Hydroxy-iso-butyrophenone. However, due to the volatile nature of VOC-3.9, the exact inhibitory concentration of VOC-3.9 against *P. aeruginosa* PAO1 needs to be double-checked using various methods.

### 3.7. Transmission Electron Microscopy (TEM) Observation

To check the morphological changes of *P. aeruginosa* PAO1 after 2-Hydroxy-iso-butyrophenone or VOC-3.9 treatment, the cells of *P. aeruginosa* PAO1 were observed by TEM (Hitachi HT7700, Tokyo, Japan). Briefly, *P. aeruginosa* PAO1 cultures were inoculated at a 1% concentration in LB medium supplemented with 0.3 mg mL^−1^ 2-Hydroxy-iso-butyrophenone or 0.3 mg mL^−1^ of VOC-3.9 at 37 °C with a rotation of 150 rpm for 16 h. Bacterial cells cultured with sterile water as controls were centrifuged at 3000× *g* for 10 min and washed twice with 0.1 M phosphate buffer solution (PBS). Cells were adsorbed on a copper mesh for 30 min and non-adsorbed cultures were rinsed away. The adsorbed mesh was dried on filter paper for 10 min. TEM was used for the final observation of the samples.

### 3.8. Proteomics Analysis

A previous study revealed that the QS systems of *P. aeruginosa* PAO1 had been activated when the OD_600_ value reached 1.0 [56]. To investigate the effects of VOC-3.9 on the QS systems and other key metabolic processes of *P. aeruginosa* PAO1, we monitored the OD_600_ values and collected the cells for proteomic analysis when the OD_600_ values of *P. aeruginosa* PAO1 were, respectively, 1.166, 1.186, and 1.078 for the control group and two experimental groups. Briefly, *P. aeruginosa* PAO1 cultures were, respectively, inoculated in LB medium supplemented with 0.375 mg mL^−1^ and 0.75 mg mL^−1^ of VOC-3.9 at 37 °C for 16 h with a rotation of 150 rpm. Bacterial cells cultured in the same condition supplemented with the same amount of sterile water were used as the control. Thereafter, the cultures were collected by centrifugation at 3000× *g* for 10 min, a twofold volume of lysis buffer (containing 8 M urea, 0.5% protease inhibitor cocktail III, 1% SDS) was added, and the lysates were fully shaken. Then, the samples were placed in a water bath at 100 °C for 30 min and broken three times on ice using ultrasound at 25% power. Finally, each sample was centrifuged at 10,000× *g* for 10 min to collect the supernatant and measure the concentration. Then, the peptide segments were digested and dissolved in a solvent containing 0.1% formic acid and 2% acetonitrile/water. They were separated using a C18 SPE column (25 cm length, 75/100 μm, Agilent, USA). The separation was carried out using a nanoElute UHPLC system (Bruker Daltonics, Billerica, MA, USA) and analyzed with a timsTOF Pro mass spectrometer. The MS/MS scan range was set from 100 to 1, 700 m/z. All of the above proteomic analyses were performed by PTMBiolabs (Hangzhou, China).

### 3.9. Protein Overexpression in P. aeruginosa PAO1

To overexpress proteins in *P. aeruginosa* PAO1, the commercial plasmid pUCP18 (Tingke, Beijing, China) was used as a starting vector. Based on our test, *P. aeruginosa* PAO1 exhibited sensitivity to gentamicin. Therefore, the original ampicillin resistance gene of pUCP18 was replaced with the gentamicin resistance gene. The Gm fragment and linearized plasmid fragment were amplified using the forward primers Gm-f and pUCP-f and the reverse primers Gm-r and pUCP18-r, respectively, and the 20 bp sequences at both ends of the linearized plasmid were included in the Gm-f/r primers as homologous regions, and then the Gm-resistant plasmid pUCP18-Gm was constructed using the homologous recombination kit (ABclonal, Wuhan, China). Subsequently, the encoding sequence of the targeting protein was amplified by the corresponding primers (*fts*-f/r) listed in Table 1. To conveniently clone the corresponding DNA fragment into the vector, appropriate endonuclease cutting sites were, respectively, introduced into two end primers. The vector and DNA fragment were cut by the same two endonucleases for 30 min and ligated together to construct the final expression vector. The resulting construct was then transformed into *E. coli* for replication and the final vector was verified by sequencing. Finally, the expression vector was transformed into *P. aeruginosa* PAO1 as described previously [57], and the corresponding assays were performed.

### 3.10. The Inhibition and Dispersion Assays of Biofilm Formation in P. aeruginosa PAO1 Treated with VOC-3.9

The anti-biofilm activity assay was performed by the method described previously [58]. *P. aeruginosa* PAO1 was cultured in LB medium overnight at 37 °C, diluted to an OD_600_ of 0.1, and divided into two groups: one group was treated with 0.375 mg mL^−1^ VOC-3.9 and one with water. Each group had three replicates, statically incubated in 96-well polystyrene plates at 37 °C for 24 h. After discarding planktonic debris, the biofilm was washed with PBS and stained with 1% crystal violet for 10 min. Subsequently, the non-adherent crystal violet was washed away using PBS. After drying, the crystal violet was dissolved in 95% ethanol. Absorbance at 595 nm was measured to quantify the biofilm.

To test the dispersion rate of VOC-3.9 on the preformed biofilm, overnight cultured *P. aeruginosa* PAO1 was similarly diluted to an OD_600_ of 0.1 using LB medium, and 200 μL of the diluted culture was incubated statically at 37 °C for 24 h. After washing away the planktonic bacterial cells, VOC-3.9 at a final concentration of 0.75 mg mL^−1^ was added. The control group was set up by adding the same amount of sterile water. The plate was then statically incubated for another 24 h, and the remaining biofilm was examined and quantified as described above. The above experiments were each repeated three times.

### 3.11. Detection of Intracellular Ferrous Ion Concentrations in P. aeruginosa PAO1

To detect intracellular ferrous ion concentrations in *P. aeruginosa* PAO1, an overnight culture was inoculated as the seed solution. The cells were then cultured in a LB medium supplemented with 0.3 mg mL^−1^ VOC-3.9 for 16 h at 37 °C with a rotation of 150 rpm at a 1% seeding concentration. Bacterial cells cultured in the same condition supplemented with the same amount of sterile water were used as the control. The cultures were centrifuged at 4 °C and 5000× *g* for 10 min. The collected cells were resuspended in PBS and were broken by ultrasonic fragmentation. The supernatant was collected at 4 °C and subjected to gradient dilution, and the protein concentration was measured at 562 nm using a BCA kit (Solarbio, Beijing, China). Simultaneously, the reaction between ferrous ions and tri-pyridyl-triazine under acidic conditions was carried out, and the concentration of ferrous ions was determined at 593 nm using the ferrous ion content detection kit (Solarbio, Beijing, China) as per the instructions. The experiments were performed in triplicate.

### 3.12. Detection of Intracellular Reactive Oxygen Species (ROS) Levels in P. aeruginosa PAO1

Intracellular ROS amounts of *P. aeruginosa* PAO1 were detected using DCFH-DA (Solarbio, Beijing, China) fluorescent probes. *P. aeruginosa* PAO1 cells were cultured overnight at 37 °C, collected, and diluted with PBS to an OD_600_ of 0.3. Subsequently, the diluted culture was incubated in the dark at 37 °C for 30 min with a working concentration of 10 μM DCFH-DA. Following incubation, the cells were washed twice with PBS to wash away the DCFH-DA that had not entered the cells. The DCFH-DA-loaded *P. aeruginosa* PAO1 cells were exposed to VOC-3.9 at concentrations of 0.3 mg mL^−1^. The above mixture exposed to the same amount of PBS was used as a control. A 100 μL solution from each sample was taken for detection at λex 488 nm and λem 525 nm every two minutes, and the experiments were performed in triplicate.

### 3.13. Statistical Analysis

All data are expressed as means ± SD. Statistical significances were analyzed by two-tailed Student’s *t*-tests using SPSS 17.0 (IBM). Differences of *p* ≤ 0.05 were considered statistically significant (* *p* ≤ 0.05, ** *p* ≤ 0.01, *** *p* ≤ 0.001). We generated the heat maps in the R software environment (R version 4.2.2). Graphical analysis was performed using the Origin 2021 software package.

### 3.14. Data Availability

The mass spectrometry proteomics data have been deposited to the Proteome Xchange Consortium (http://www.ebi.ac.uk/pride (accessed on 20 January 2024)) with the dataset identifier PXD045249.

## 4. Conclusions

**Figure 7:**
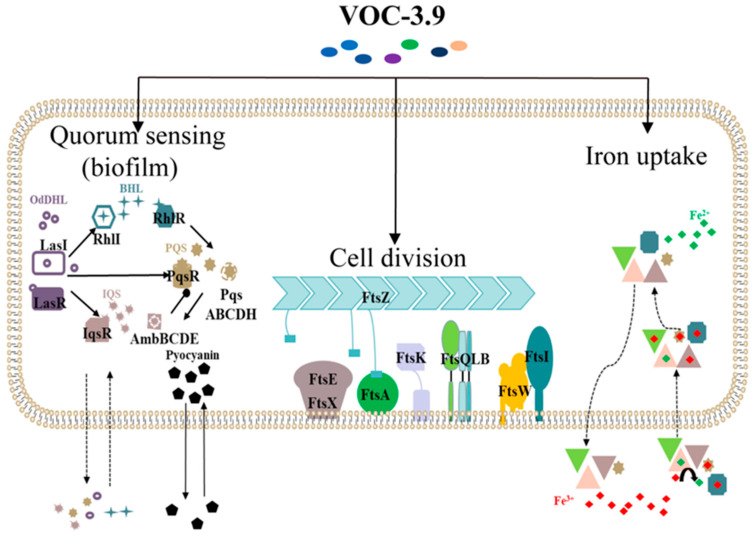
A proposed model of VOC-3.9 inhibiting the growth of *P. aeruginosa* PAO1. This model describes three major physiological processes (including QS systems associated with biofilm formation/dispersion, cell division, and iron uptake processes) in *P. aeruginosa* PAO1 that are primarily affected by VOC-3.9. The diagram presents four QS-system signal networks in *P. aeruginosa* PAO1 and their respective autoinducers. The dashed and solid lines represent different transmembrane transport processes. The connection between Fts-related proteins with sPG synthesis is indicated. Various pathways for extracellular Fe^3+^ uptake by *P. aeruginosa* PAO1 are shown, including siderophores: pyoverdine and pyochelin (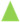), Heme uptake pathway: Has, Phu systems (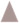), Feo uptake pathways (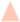), and heterologous uptake pathway (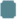).

Overall, the volatile organic component VOC-3.9 produced by the Gram-negative bacterium *Spongiibacter nanhainus* CSC3.9 from the deep sea effectively inhibits environmental and human pathogenic bacteria as well as agricultural pathogenic fungi, especially *P. aeruginosa* PAO1. Based on the combination of microscopic, proteomic, and biochemical data shown in this study, we propose the overall growth inhibition pathways in *P. aeruginosa* PAO1 treated by VOC-3.9 as follows (Figure 7). First, VOC-3.9 induces abnormal cell division by interfering with the expression of essential division proteins associated with septal peptidoglycan synthesis. Furthermore, VOC-3.9 impedes bacterial quorum sensing systems, thereby hindering biofilm formation and promoting biofilm dispersion processes. Lastly, VOC-3.9 disrupts the iron uptake systems, leading to reduced enzymatic activity associated with key metabolic processes (e.g., ROS elimination). Future efforts will be required to identify the exact components directing antibacterial function and study their synergistic effects and mechanisms both in vitro and in vivo.

## Figures and Tables

**Table 1:** Primers used for protein overexpression in *P. aeruginosa* PAO1.

Prim Name	Sequence (5′-3′)
*ftsZ*-f	TAC**GAATTC**AATGTTTGAACTGGTCGATAACA (*Eco*RΙ, underlined)
*ftsZ*-r	AGA**GGATCC**TCAATCGGCCTGACGAC (*Bam*HΙ, underlined)
*ftsL*-f	TAC**GAATTC**AATGAGCCGTCTCTTCGTCAAG (*Eco*RΙ, underlined)
*ftsL*-r	AGA**GGATCC**TCATGGCGCCACCATCCT (*Bam*HΙ, underlined)
*ftsB*-f	TAC**GAATTC**TTGAGGTTACGTAGCCCCTACT (*Eco*RΙ, underlined)
*ftsB*-r	AGA**GGATCC**TCACTTGGCGAGCTGGTAGA (*Bam*HΙ, underlined)
*ftsI*-f	TAC**GAATTC**ATGAAACTGAATTATTTCCAGGGCG (*Eco*RΙ, underlined)
*ftsI*-r	AGA**GGATCC**TCAGCCACGCCCTCCTTTTG (*Bam*HΙ, underlined)
*ftsE*-f	TAC**GAATTC**ATGATCCGCTTCGAGCAGGT (*Eco*RΙ, underlined)
*ftsE*-r	AGA**GGATCC**TCAGGCCTCATCCTCACGGTCA (*Bam*HΙ, underlined)
*ftsQ-f*	TAC**GAATTC**ATGAATGGCGTACTGCTCCG (*Eco*RΙ, underlined)
*ftsQ-r*	AGA**GGATCC**TCACTGCACGGCGCTGG (*Bam*HΙ, underlined)
Gm-f	AATATTGAAAAAGGAAGAGTATGTTACGCAGCAGCAACGA
Gm-r	GAGTAAACTTGGTCTGACAGTTAGGTGGCGGTACTTGGGT
pUCP18-f	CTGTCAGACCAAGTTTACTCATATATACTT
pUCP18-r	ACTCTTCCTTTTTCAATATTATTGAAGCAT

## Data Availability

The data presented in this study are available on request from the corresponding author.
